# Recall DNA methylation levels at low coverage sites using a CNN model in WGBS

**DOI:** 10.1371/journal.pcbi.1011205

**Published:** 2023-06-14

**Authors:** Ximei Luo, Yansu Wang, Quan Zou, Lei Xu

**Affiliations:** 1 School of Electronic and Communication Engineering, Shenzhen Polytechnic, Shenzhen, Guangdong, China; 2 Institute of Fundamental and Frontier Sciences, University of Electronic Science and Technology of China, Chengdu, Sichuan, China; 3 Yangtze Delta Region Institute (Quzhou), University of Electronic Science and Technology of China, Quzhou, Zhejiang, China; CANADA

## Abstract

DNA methylation is an important regulator of gene transcription. WGBS is the gold-standard approach for base-pair resolution quantitative of DNA methylation. It requires high sequencing depth. Many CpG sites with insufficient coverage in the WGBS data, resulting in inaccurate DNA methylation levels of individual sites. Many state-of-arts computation methods were proposed to predict the missing value. However, many methods required either other omics datasets or other cross-sample data. And most of them only predicted the state of DNA methylation. In this study, we proposed the RcWGBS, which can impute the missing (or low coverage) values from the DNA methylation levels on the adjacent sides. Deep learning techniques were employed for the accurate prediction. The WGBS datasets of H1-hESC and GM12878 were down-sampled. The average difference between the DNA methylation level at 12× depth predicted by RcWGBS and that at >50× depth in the H1-hESC and GM2878 cells are less than 0.03 and 0.01, respectively. RcWGBS performed better than METHimpute even though the sequencing depth was as low as 12×. Our work would help to process methylation data of low sequencing depth. It is beneficial for researchers to save sequencing costs and improve data utilization through computational methods.

## Author summary

DNA methylation has a major impact on gene regulation. WGBS is the gold standard for investigating the DNA methylation. The DNA methylation level of the sites with low coverage are often not accurate in WGBS datasets. Therefore, we proposed a method based on the CNN model to perform DNA methylation level interpolation for specific sites and named this method as RcWGBS. RcWGBS did not rely on other omics data or other cross-sample data. It only used the sites with sufficient coverage contained in the target WGBS dataset for model training to obtain parameters. Then, the trained model can be used to predict the DNA methylation level of sites with low coverage. Our analyses showed that RcWGBS could recalibrate the methylation level of some CpGs with insufficient coverage. It is suggested that our research could benefit the WGBS datasets with insufficient sequencing coverage. RcWGBS is implemented as an R-packages. It is efficient and convenient and does not need other WGBS or omics data.

This is a *PLOS Computational Biology* Methods paper.

## Introduction

Cytosine methylation is a widely conserved epigenetic mark with very important roles in many biological regulatory processes such as cell differentiation, development, and many diseases [1–7]. It is a covalent chemical modification that can alter and downregulate gene expression by stably affecting transcription factor binding [8–11]. Most DNA methylation occurs in the CpG dinucleotides [12–18]. Whole-genome bisulfite sequencing (WGBS) is a next-generation sequencing method that can detect and quantify DNA methylation at genome-wide base resolution [19–21]. The application of this technology has been instrumental in dissecting the molecular pathways by which DNA methylation controls gene expression dynamics by steering transcription factors. However, deep inter-and intraspecific WGBS measurements remain cost prohibitive, particularly for species with large genomes [22–24]. The NIH Roadmap Epigenomics Project currently recommends that the WGBS have at least 30× coverage with two replicates (http://www.roadmapepigenomics.org/protocols). Many published methylomes have therefore been sequenced far below saturation (i.e., a large number of cytosines in the genome are not covered, or the coverages are less than 3). Even if the coverage is sufficient, there are still many sites with coverage of less than 3. For example, the combined coverages of the WGBS data of GM12878 and H1-hESC in the ENCODE [25] database are 59.58X and 54.08×, respectively. However, approximately 4% of the CpG sites have coverages ≤ 3. It would be more serious accumulation effective if multiple groups of WGBS data were combined for further analysis [26, 27].

There is currently much interest in calling DNA methylation in single cells and in nanotechnology [28, 29]. In single-cell methylomes, there are sites not covered by any reads in some single cell. The DNA methylation status of other cells and DNA sequenced can be used as the features of the deep learning model to predict the methylation status [30–32]. However, in traditional bulk WGBS data, calling DNA methylation also has the problem of insufficient coverage. The lower the coverage of WGBS is, the lower the accuracy of the DNA methylation level. Interpolation, smoothing, and missing-value filling methods have been proposed to solve this problem, including METHimpute [33]. The HMM model was used to interpolate the DNA methylation level by taking all reads of CpG sites and the number of methylated reads of the entire genome as inputs. This method has been applied to plant genomes and has proved to be effective. This method requires the input of the whole DNA methylation chain for model training and prediction. In addition, DNA methylation has sequence characteristics, such as CpG-rich regions that are mainly unmethylated with a C+G content greater than 50% [34–37]. A number of studies have been conducted to predict the methylation status of CpG based on flanking sequences and TF binding motifs [37–39]. Wang et al. proposed DeepMethyl based on sequence and Hi-C data to predict the methylation state [40]. Wu et al. and Zhou et al. also proposed methods to predict DNA methylation status based on SVM using DNA sequences on their own set of benchmark data [41–47]. Only using DNA sequences to predict DNA methylation status can obtain good prediction results, but this approach can only be applied to specific datasets. In practical applications, although DNA sequences are consistent in different cells, DNA methylation levels are different, so other dynamic characteristics are required to predict DNA methylation levels dynamically [48–50]. Related methods have limited predictions on methylation states or are based on other omics data [42, 51–54]. Only METHimpute can be used to dynamically impute missing DNA methylation levels independent of other omics data. METHimpute uses DNA methylation level chains. Here, we found that DNA sequence characteristics and methylation levels on flanking regions can both be used for imputation. The WGBS sequencing coverage of the sites to be predicted is low, but the coverage of its flanking sites is available to predict the methylation of low-coverage sites.

**Fig 1:**
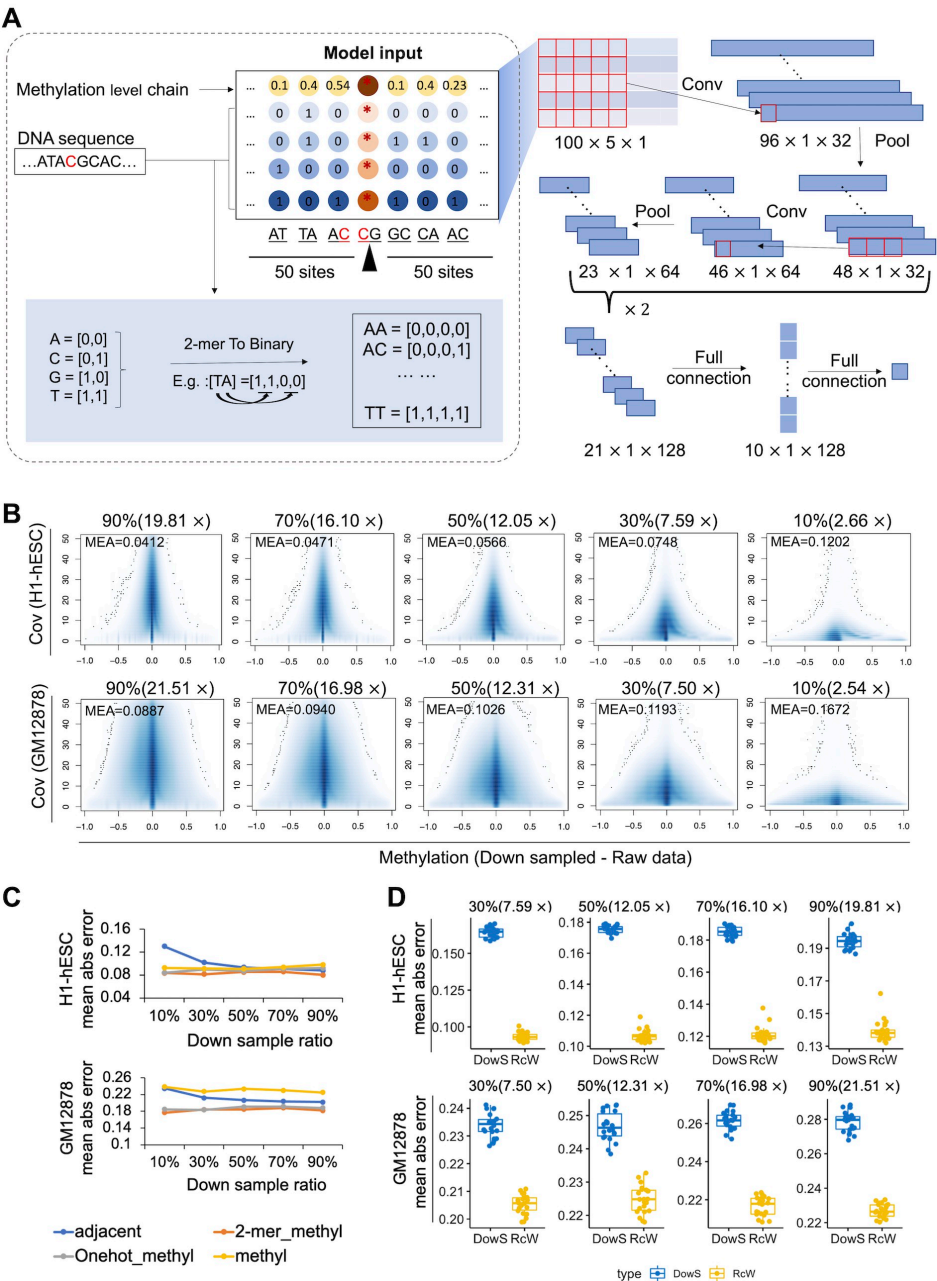
The structure of the RcWGBS model and results by using the RcWGBS in H1-hESC and GM12878 datasets. **(A)** The structure of the RcWGBS model. The DNA sequence and the DNA methylation levels were used as the input features. The 2-mer coding method was used to encode flanking DNA sequences centered on the sites with 50 bp upstream and downstream. Finally, the input feature of RcWGBS was a data matrix with a length of 100, a width of 5, and a height of 1. **(B)** The lower the coverage, the greater the difference between the DNA methylation level in the down-sampling and the original data. *MEA* means the mean absolute error. **(C)** Difference between predicted DNA methylation level and original DNA methylation level under different features. Y-axes represented the mean absolute error. **(D)** The mean absolute error of the imputed methylation calls in down-sampled H1-hESC and GM12878 data could be reduced. The blue dots represented the difference between the DNA methylation level of the down-sampled and the unsampled original dataset. While the yellow dots represented the difference between the DNA methylation level after RcWGBS interpolation and the unsampled original data. A total of 22 groups of data were compared here.

In this study, we downsampled the original data and compared the DNA methylation after sampling with the original DNA methylation. It was found that the lower the coverage was, the greater the difference in DNA methylation level (as shown in Fig 1B). To maximize the information contained in WGBS data and to facilitate cost-effective sequencing decisions for future studies, we developed RcWGBS, a convolutional neural network (CNN)- based imputation algorithm for the construction of base pair resolution methylomes from WGBS data. The unique feature of this algorithm is its ability to impute the methylation level of cytosines with missing or uninformative coverage, thus yielding complete methylomes even with low-coverage WGBS datasets. Indeed, we downsampled the WGBS data of two cell lines and then used RcWGBS to speculate the DNA methylation data with low coverage after sampling. Then the DNA methylation level of the speculated WGBS was compared with the raw unsampled values. This method can effectively improve the accuracy of the DNA methylation level with low coverage.

## Results

### Conceptual overview

WGBS is an NGS-based method in which DNA is treated with sodium bisulfite before sequencing to convert unmethylated cytosines into uracils and ultimately into thymines during PCR amplification. Hence, a cytosine in a bisulfite-treated read that maps to a cytosine in the reference genome provides evidence for methylation, while a thymine that maps to a cytosine does not. The DNA methylation level is defined as the number of methylated reads covering a specific site divided by the total number of reads. At a specific CG site, the methylation level is defined as the number of reads with methylated cytosines divided by the total number of reads covering that site. In the actual experiment, there were sites that were not fully covered, so the DNA methylation level could not be calculated effectively. To overcome these limitations, we developed RcWGBS, a CNN-based approach to impute missing values from WGBS. The binding sites of transcription factors are affected by DNA methylation. Therefore, we can assume that in a cell state, the level of DNA methylation is related to the DNA sequence pattern. In addition, the distribution of DNA methylation has the characteristics of spatial distribution [55, 56]. RcWGBS takes methylation level chains from Bismark or other apps as input by integrating the DNA sequence information. The outputs are recalibrated methylation levels between 0 and 1 for every cytosine in the genome.

For the DNA sequence, we selected the sequence centered on the sites with 50 bp upstream and downstream. In this study, we tested two coding methods, one-hot and 2-mer [57]. The 2-mer method carries more sequence information than the one-hot method, so it performs better than the one-hot method. Therefore, in this study, the 2-mer coding method was used to encode DNA sequences. Sixteen kinds of 2 bp subsequences can be composed of 4 bases, which can be expressed by the numbers of 0–15. Then the decimal data are binarily converted, and finally, they can be expressed as a vector with a length of 4 and only containing 0 and 1 (as shown in Fig 1A). Since the DNA methylation level of adjacent regions is consistent, the DNA methylation level of the region adjacent to the predicted site can also be used as a feature. Here, the DNA methylation levels of 50 sites upstream and downstream of the site to be predicted were used. Finally, the input feature of the model was a data matrix with a length of 100, a width of 5, and a height of 1. The RcWGBS was based on a convolutional neural network. The model performs the first feature extraction through a 5×5 two-dimensional convolution kernel. After pooling, two one-dimensional convolutions are performed again to enhance feature extraction [58, 59]. Then after the full connection, a final output value of 0–1 was used to infer DNA methylation. The overall structure of the RcWGBS model is shown in Fig 1A.

### Feature combination and selection of DNA sequence representation

For the data used to build the model, we first downsampled the readings after WGBS alignment of the GM12878 and H1-hESC datasets and sampled 90%, 70%, 50%, 30%, and 10%, respectively [60]. The coverage after sampling is shown in Table 1, and the minimum coverage was 2.54×(per cytosine, double-stranded). We used DNA methylation chain, DNA methylation chain combined with DNA sequence encoded by one-hot, and DNA methylation combined with DNA sequence encoded by 2-mer as model input. In addition, in experiments, the average methylation level on both sides of adjacent sites is often used as the methylation level of sites to be estimated. We compared the predicted results of these three combined features input into the CNN model with the average methylation levels on both sides of the adjacent sites. The mean absolute error between the predicted results and the results of unsampled data was used as the evaluation index. The mean absolute error (*MAE*) is defined as:

$$MAE=\frac{\sum_{i=0}^{N}\left|m_{i}-m_{i}^{\prime }\right|}{N}$$

where *m*_*i*_ and $m_{i}^{\prime }$ represent the true and predicted values of DNA methylation, respectively.

**Table 1:** Coverage of down-sampled data.

	H1_hESC	GM12878
Raw_data	54.08	59.58
90%	19.81	21.51
70%	16.10	16.98
50%	12.05	12.31
30%	7.59	7.50
10%	2.66	2.54

We selected the training data based on the statistics of coverage. DNA methylation levels at CpG sites with coverage between the median and the third quartile are considered relatively accurate. These loci were selected for the training model. In the process of selecting the feature representation, 100,000 sites were used as the training set. The independent test dataset was 100,000 sites from other sites that were selected randomly and not included in the training set. The results are shown in Fig 1C. We found that DNA sequence features improved prediction significantly versus using only the neighbor DNA methylation levels. The DNA sequence encoded by the 2-mer combined with the DNA methylation chain as an input feature can obtain the best results. Finally, the input features of the model were DNA sequences and DNA methylation chains represented by 2-mers. All of the computer programs and scripts can be downloaded from https://github.com/TracyHIT/RcWGBS/.

### Imputation of the downsampled H1-hESC and GM12878 methylomes

To demonstrate the performance of the RcWGBS, we analyzed WGBS data with different coverage. Additionally, using the coverage statistics, 100,000 CpGs with coverages are at the median, and the third quartile were randomly selected as the training set. Then, other sites with insufficient coverage or less than three were interpolated. We found that RcWGBS could produce high-quality interpolation and correction for methylation calls with different coverages. In the downsampled data, we counted the changes in the methylation levels of CpG sites with coverage less than three but with coverage greater than ten in the unsampled data. Indeed, on CpG sites with insufficient coverage, the *MAE* of the imputed methylation calls in downsampled H1-hESC and GM12878 data could be reduced. As shown in Fig 1D, each point represents the *MAE* of each chromosome. Here only the sites with insufficient coverage or less than three were counted. In the H1-hESC dataset, the mean absolute error in the DNA methylation level between downsampled data and the original data was greater than 0.158, while in the GM12878 dataset, the mean absolute error was greater than 0.226. This difference was significantly reduced after using RcWGBS in the two datasets. Among the sites to be compared (sites with insufficient coverage lower than three in the down-sampled data were counted), with the increase in the sequencing depth, the difference between the coverage in the down-sampled data and the original data became larger. So, the error between the DNA methylation level obtained from the sampled data and the original data gradually increased (as shown by the blue dots in Fig 1D). By RcWGBS prediction, *MAE* had shrunk by 0.037 and 0.065 on average, compared with downsampled dataset in GM12878 and H1-hESC, respectively. Other results were better than these, collectively proving the effectiveness of RcWGBS.

### Comparison with METHimpute and BSmooth

**Fig 2:**
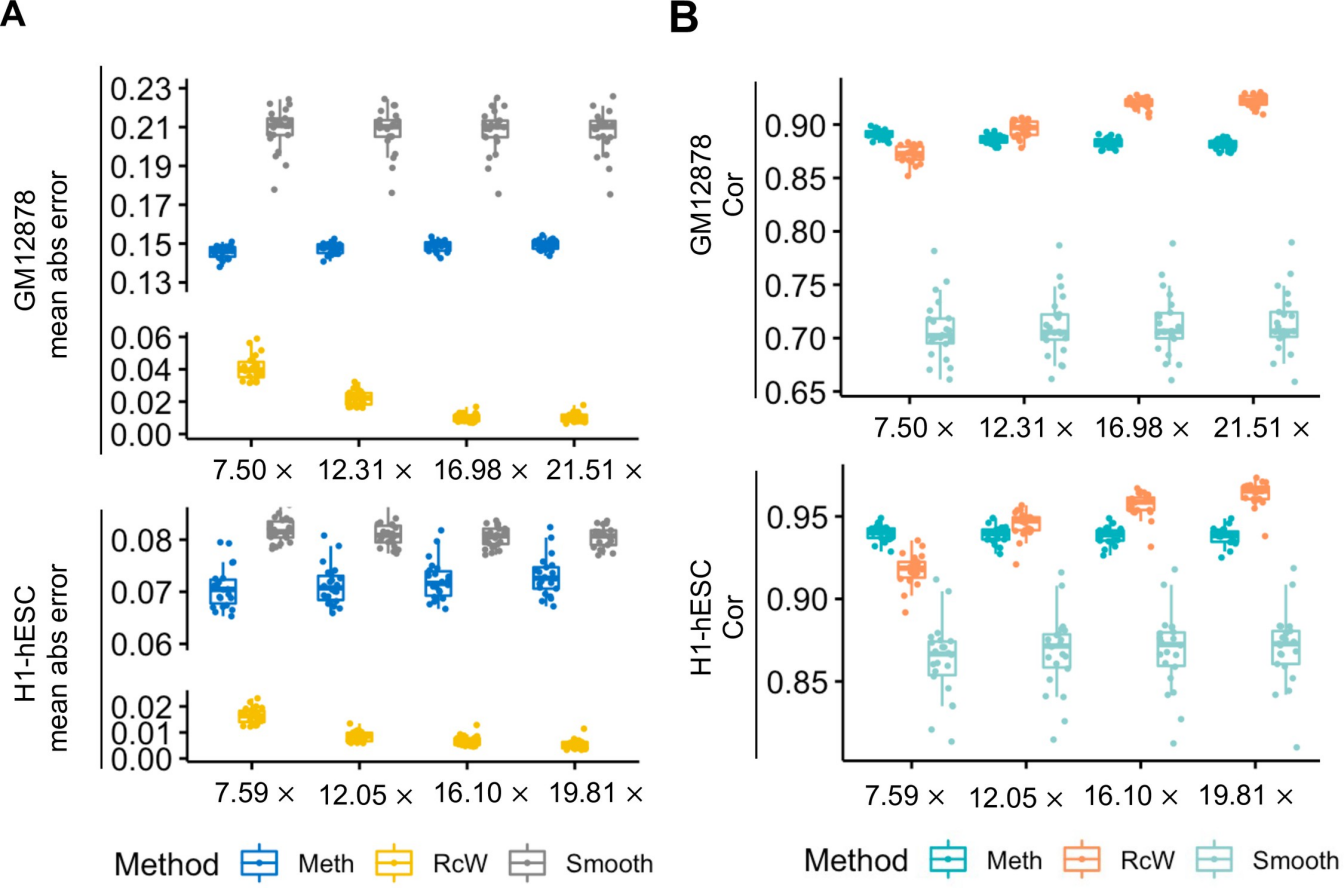
Comparison with METHimpute and BSmooth. **(A)** The mean absolute error of the DNA methylation level between raw unsampled data and predicted values from RcWGBS, METHimpute, and BSmooth, respectively. **(B)** The pearson’s correlation coefficient between the raw unsampled data and predicted values from RcWGBS, METHimpute, and BSmooth, respectively.

For WGBS data, METHimpute and BSmooth have been proposed. METHimpute is a method based on the HMM model to infer DNA methylation from insufficient sequencing. BSmooth is the popular smoothing-based method. We used METHimpute and BSmooth to interpolate the methylation levels of the downsampled data at base pair resolution. The methylated read number and the total read number at every site were input into METHimpute and BSmooth. METHimpute assumes that there are two distributions of DNA methylation levels and re-estimates each site’s methylation level. In the downsampled data, the DNA methylation level at the sites with high coverage was more accurate. Therefore, the sites with sufficient coverage were used to train the CNN model. Only the sites with low coverage were re-estimated in RcWGBS. As shown in Fig 2A and listed in S1 Table, in the H1-hESC dataset, the mean absolute error of the DNA methylation level between the original data and downsampling data estimated by METHimpute and BSmooth was greater than 0.05, while in GM12878, the mean absolute error was greater than 0.14 (as listed in S2 Table). This difference was significantly reduced after using RcWGBS compared with using METHimpute. By RcWGBS, the mean absolute errors were reduced to less than 0.01 and 0.05 in the H1-hESC and GM12878 datasets, respectively. To evaluate prediction accuracy, we also calculated the Pearson’s correlation coefficient between the raw unsampled data and predicted values using RcWGBS and METHimpute. We found that when the coverage was too low, the correlation coefficient between the predicted value and the true value of RcWGBS was reduced and lower than that of METHimpute (as shown in Fig 2B). In the H1-hESC and GM12878 datasets, when the coverages were approximately 12.05 and 12.31, respectively, the correlation coefficients between the predicted value of RcWGBS and the unsampled value were higher than those of METHimpute and BSmooth (as listed in S3 Table and S4 Table). With increasing coverage, the accuracy of RcWGBS increased. The correlation coefficient of METHimpute was relatively stable. However, when the coverage was high, the correlation coefficient was lower than the prediction result of RcWGBS. These results prove that the RcWGBS is better than METHimpute.

## Discussion

WGBS is considered to be the "gold standard" for single-base resolution measurement of DNA methylation levels. However, WGSB often requires high sequencing depth. Some sites with insufficient coverage are observed in WGBS data. The DNA methylation levels of these sites were often not accurate [61]. These sites would affect further analysis in subsequent analysis, such as calling differential methylation sites and DNA methylation biomarkers for the disease. Therefore, it’s very important to obtain accurate DNA methylation levels on sites with insufficient coverage. A large number of studies have shown that DNA methylation level has spatial distribution characteristics and DNA sequence characteristics, which is consistent with the DNA methylation level of flanking sites [34–37]. Therefore, a large number of methods have been proposed to predict the DNA methylation state or level. But most of them need the other omics data [42, 51–54]. In addition, there are some methods that only use DNA sequences to predict DNA methylation status on the benchmark dataset [37–39]. As DNA methylation is dynamic, these prediction methods without any dynamic data seems unreasonable. Therefore, many methods cannot effectively predict DNA methylation levels in low-coverage WGBS datasets.

In 2018, the METHimpute method was proposed [33]. It used HMM model only based on DNA methylation characteristics. In this work, RcWGBS combined DNA sequence and DNA methylation information and took advantage of the CNN model in information extraction. It used the DNA sequence and DNA methylation levels on both sides of the site as features. For the RcWGBS, it was not necessary to provide the entire DNA methylation data chain. When predicting minority points, it only needs to provide DNA methylation and DNA sequence on both sides of the site to be predicted by using RcWGBS. Through the application in the H1-hESC and GM12878 datasets, we proved that the RcWGBS performed better than METHimpute.

In addition, in the METHimpute model, only two states of DNA methylation were considered. Therefore, the interpolated DNA methylation level is mainly distributed in the two regions close to 0 and 1, resulting in some DNA methylation near 0.5 being overestimated or underestimated. Although the correlation coefficient is higher than RcWGBS in an extremely low coverage profile, the *MAE* of METHimpute is lower than RcWGBS. In the RcWGBS model, when a large number of sites (>1 million) need to be predicted, the pre-processing time of RcWGBS is large, and the upstream and downstream DNA sequences of the sites to be predicted need to be extracted. In order to solve this problem, the reference genome can be used to extract the sequences on both sides of all CpG sites in advance. In the R package download link provided in this article, the data matrix of 50bp sequences upstream and downstream on both sides of all CpG sites of GRch38 has been provided.

It is noteworthy to mention that the applicability of RcWGBS in single-cell sequencing using WGBS data has been further investigated. By modifying the loss function and optimization method during the model training process, RcWGBS can be adapted into a prediction classification model, enabling accurate prediction of DNA methylation in single-cell WGBS.

## Materials and methods

### Downsampling data preparation

The WGBS sequencing data used in this study were downloaded from ENCODE [25]. For the H1-hESC dataset, files numbered ENCFF003FWN and ENCFF546TLK were downloaded. For the GM12878 dataset, files numbered ENCFF857QML and ENCFF681ASN were downloaded. Then the four files were randomly down-sampled to different degrees by samtools, such as 90%, 70%, 50%, 30%, and 10%. Randomly sampled the reads of the raw data directly. Downsampling the pair-end sequencing files cannot guarantee that the reads were sampled in pair-end. Therefore, the changes in coverage and adoption ratio were different. The independent test dataset was constructed by covering the sites selected as the training data. The training sites were randomly sampled from the whole genome. It ensured the uniformity of the training sample on the whole genome. For the documents produced after sampling, Bismark [62] was used to extract the DNA methylation level. Then, the two repetitions in the experiment were merged by combining the numbers of methylated and unmethylated reads in the two repetitions. Since CpG is symmetric, the DNA methylation levels on the negative chain and positive chain are combined. Methylation values for each CpG site were quantified by m, which is the fraction of methylated reads over the total reads:

$$m=\frac{Meth\_C}{Meth_{C}+Unmeth_{C}+100}$$

where *Meth*_*C* and *Unmeth*_*C*_ represent the methylated and unmethylated reads called by bismark.

### CNN for methylation calling

A CNN model with multiple convolutional and pooling layers and two fully connected hidden layers were used to extract features from high-dimensional inputs. The whole model was convoluted three times and pooled after each convolution. The kernel of the first convolution calculation was 5×5 and the step size was 1. The kernel function of the second and third convolution calculations was 3×1 and the step size was 1. The input was a 100bp long DNA sequence and DNA methylation chain centered on the target CpG site. The DNA methylation chain consisted of the methylation levels of 100 CpGs upstream and downstream. 100 2-mers can be generated from DNA sequence. The step size was set as 1, There was an overlay of one base between two adjacent 2mers. One-hot and 2-mer coding methods were used for DNA sequence representation. In the one-hot coding process, [[0,0,0,1], [0,0,1,0], [0,1,0,0], [1,0,0,0]] were used to for encoding the four different nucleotides of A, C, G and T. In the 2-mer coding process, 0–3 binary representations were used for the four nucleotides, and then 2mer directly splices the corresponding binary coding of the two bases. As Fig 1A shows, this encoding method is equivalent to encode AA, AC,…, TT corresponding to the binary encoding of 0,1,…, 15. Finally, for a specific target site, the input was a matrix *s* with the 5 rows, 100 columns and 1 channel. *s* was first transformed by a 2d-convoluntional layer, which computed the activations *a*_*fi*_ of a convolutional filter *f* at every position *i* in a matrix *s*:

$$a_{f,i}=ReLU(\sum_{l=1}^{L}\sum_{d=1}^{D}w_{f,l,d}s_{i+l,d})$$

Here the *w*_*f*_ was the weight matrix of convolutional filter f of length L and wide D. The input channel was one. The input row number was 5. The D was set as 5. The first convolution kernel was 5×5. Here the L was set as 5. A pooling layer was used to summarize the activations of p adjacent neurons by their maximum value P:

$$P=max\;(a_{f,i},\ldots ,a_{f,i+p-1})$$

Here p was set as 2 or 3. After the first convolution and pooling, the 2d-convolutional layer degenerated to 1d-convolutional layer. Two fully connected layers were used in the model. The first fully connected layer converted a matrix with a size of 21×1×128 into a matrix with a size of 10×1×128, and the second fully connected layer mapped the matrix to the final predicted value of DNA methylation. This model was implemented in R language. The model was built using the “keras” package. The loss function of the CNN model was the mean squared error (*MSE*):

$$MSE(m,m^{\prime })=\frac{\sum_{i=1}^{n}{\left(m_{i}-m_{i}^{\prime }\right)}^{2}}{n}$$

where *m*_*i*_ and $m_{i}^{\prime }$ were the experimental and predicted DNA methylation levels of the *i*th CpG site, respectively. The model parameters were fitted with the Adam algorithm. The DNA methylation of sites with sufficient coverage was more accurate. When training the data used in the model, sites with appropriate coverage were used. We randomly selected 100,000 CpGs with coverage at the median and the third quartile as the training set. Epoch parameters are essential in the process of model training to prevent overfitting. Therefore, the validation dataset was divided from the training set during training. In each round, the losses of the training set and the verification set were calculated. In addition, the mean absolute errors (*MAE*) of the training set and verification sets were also calculated. The smaller the value was, the better the fitting effect. It was convenient for users to intuitively the optimal parameters intuitively. After each round of training, a visual figure of the *MSE* and *MAE* was output. Then, the optimal epoch parameters were set according to the figure.

### Comparison with METHimpute and BSmooth

The METHimpute method used the number of reads covered by methylation and the total number of covers as inputs. According to METHimpute’s user guide, the prediction results of points with a posteriormax greater than 0.98 were selected. In the BSmooth, default parameter was used. The predicted methylation level of the CpG point was compared with the DNA methylation level of the original WGBS data. We calculated the mean absolute error and Pearson’s correlation coefficient to evaluate the prediction accuracy:

$$r_{m,m^{\prime }}=\frac{\sum_{i=1}^{n}\left(m_{i}-\bar{m})(m_{i}^{\prime }-\bar{m^{\prime }}\right)}{(n-1)\times \sigma_{m}\times \sigma_{m^{\prime }}}$$

where *m*_*i*_ and $m_{i}^{\prime }$ were the experimental and predicted DNA methylation levels of the *i*th CpG site, respectively. $\bar{m}$ and $\bar{m^{\prime }}$ were the means of the experimental and predicted methylation levels. *σ*_*m*_ and *σ*_*m*′_ were the standard deviations of *m*_*i*_ and $m_{i}^{\prime }$.

## Supporting information

S1 Table The mean absolute error of the DNA methylation level between raw unsampled data and predicted values from RcWGBS, METHimpute and BSmooth in GM12878. (XLSX)

S2 Table The mean absolute error of the DNA methylation level between raw unsampled data and predicted values from RcWGBS, METHimpute and BSmooth in H1-hESC. (XLSX)

S3 Table The pearson correlation coefficient of the DNA methylation level between raw unsampled data and predicted values from RcWGBS, METHimpute and BSmooth in GM12878. (XLSX)

S4 Table The pearson correlation coefficient of the DNA methylation level between raw unsampled data and predicted values from RcWGBS, METHimpute and BSmooth in H1-hESC. (XLSX)
